# Downstream invasive coronary procedures following PET Versus SPECT myocardial perfusion imaging: a propensity-matched analysis

**DOI:** 10.1007/s00259-026-08062-3

**Published:** 2026-07-11

**Authors:** Chadi Tabaja, Issam Motairek, Bryan Abadie, Laura Young, Venu Menon, Wael A. Jaber

**Affiliations:** Department of Cardiovascular Medicine, Cleveland Clinic, Cleveland, OH USA

## Abstract

**Methods:**

We performed a retrospective analysis using the TriNetX Research Network. Adults undergoing PET or SPECT MPI without prior coronary revascularization were identified. The primary endpoint was invasive coronary angiography (ICA) within 90 days, with percutaneous coronary intervention (PCI) as a secondary endpoint. Propensity score matching (1:1) was performed to adjust for baseline differences.

**Results:**

A total of 33,822 propensity-matched patients were included (PET n=16,911; SPECT n=16,911). At 90 days, ICA occurred less frequently after PET compared with SPECT (6.0% vs 6.8%; RR 0.89, 95% CI 0.82-0.97; p=0.005). Conversely, PCI was more frequent following PET (0.9% vs 0.4%; RR 2.00, 95% CI 1.51-2.65; p<0.001), indicating a higher angiography-to-PCI procedural yield. These findings were consistent across sex-based subgroups.

**Conclusion:**

In a large real-world cohort, PET MPI was associated with fewer downstream invasive coronary angiograms and a higher procedural PCI yield compared with SPECT. These findings suggest that PET may improve selection of patients for invasive evaluation and enhance efficiency of downstream care.

Myocardial perfusion imaging (MPI) is a cornerstone of noninvasive evaluation for suspected coronary artery disease (CAD). Single-photon emission computed tomography (SPECT) remains the most used modality owing to its diagnostic and prognostic historical value, cost-effectiveness, broad availability, and flexibility to be performed with either exercise or vasodilator testing [1]. In contrast, positron emission tomography (PET) offers higher spatial resolution, built-in attenuation correction, real-time stress imaging, and quantitative assessment of myocardial blood flow and flow reserve, translating into superior sensitivity and specificity as well as reader/interpretation confidence compared to SPECT. Accordingly, major American and European guidelines endorse the preferential use of PET over SPECT when available. More recently, the American Society of Nuclear Cardiology issued an updated position statement recommending PET MPI as the preferred modality for patients who meet criteria for MPI [2].

The higher upfront cost of PET is frequently cited as a barrier to its broader adoption in noninvasive CAD evaluation. However, this perspective often focuses on test acquisition cost rather than the total cost of care across the diagnostic pathway. If improved diagnostic performance leads to more selective referral for invasive coronary angiography (ICA) and revascularization, PET may mitigate cumulative downstream expenditures associated with unnecessary procedures. Although prior studies have established the diagnostic advantages of PET, less is known about how imaging modality influences downstream invasive coronary utilization in contemporary real-world practice. Accordingly, we sought to evaluate the association between PET versus SPECT MPI and subsequent ICA and revascularization in a large real-world cohort, with a prespecified secondary analysis examining sex-based differences.

## Methods

We conducted a retrospective observational study using the TriNetX Research Network, a federated electronic health record database comprising data from multiple U.S. health care organizations. *Patients (≥ 18 years) undergoing MPI with either PET or SPECT for the evaluation of suspected coronary artery disease or chronic coronary syndrome were identified. Patients with prior coronary revascularization were excluded*.

The index date was defined as the date of the MPI study. To account for baseline differences, 1:1 propensity-score matching was performed using greedy nearest-neighbor matching without replacement and a caliper of 0.1 pooled standard deviations. *Covariates included age*,* sex*,* race*,* hypertension*,* hyperlipidemia*,* diabetes mellitus*,* obesity*,* smoking history*,* chronic kidney disease*,* heart failure*,* atrial fibrillation*,* peripheral vascular disease*,* and cerebrovascular disease. Covariate balance was assessed using standardized mean differences*,* all of which were < 0.10 after matching.* The study was exempt from Institutional Review Board approval because of use of aggregate, deidentified data from the research network database.

The primary endpoint was invasive ICA within 90 days MPI, with percutaneous coronary intervention (PCI) as the secondary endpoint. Two complementary analyses were performed: (1) to directly compare imaging modalities, 1:1 propensity-score matching was performed, yielding a matched cohort of 33,822 patients (PET *n* = 16,911; SPECT *n* = 16,911), which served as the primary analytic population for modality-based comparisons; (2) sex-based differences in downstream invasive utilization were evaluated in an unmatched cohort comprising 12,742 patients undergoing PET and 929,648 patients undergoing SPECT, with comparisons made between men and women within each imaging modality.

## Results

The mean age of the matched cohort was 63.3 years (PET) and 63.9 years (SPECT).

**Fig. 1:**
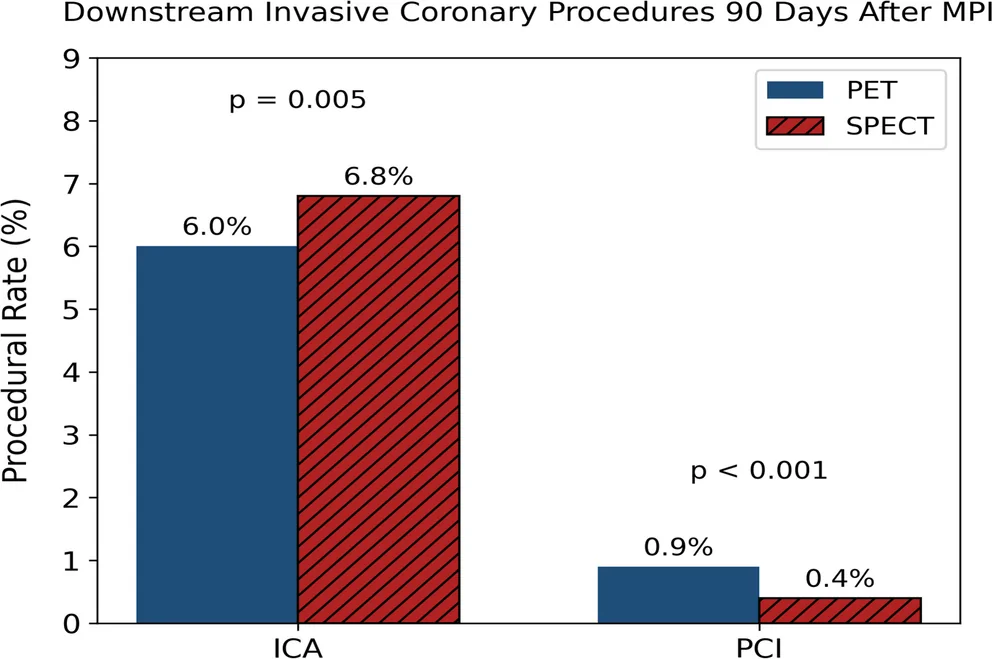
Rates of invasive coronary angiography (ICA) and percutaneous coronary intervention (PCI) within 90 days of PET (blue bar) or SPECT (red bar) myocardial perfusion imaging (MPI)

At 90 days, ICA occurred less often after PET compared with SPECT (6.0% vs. 6.8%; risk ratio [RR] 0.89, CI 0.82–0.97; *p* = 0.005). Conversely, PCI was more frequent after PET (0.9% vs. 0.4%; RR 2.00, 95% CI 1.51–2.65; *p* < 0.001. A summary of the results is shown in Fig. 1.

Across both modalities, men were more frequently referred for ICA and more likely to undergo PCI compared with women; PET: (7.2% vs. 5.4%; RR 1.33, CI 1.16–1.52) and PCI (1.2% vs. 0.6%; RR 1.81, CI 1.23–2.64); SPECT: (7.1% vs. 5.6%; RR 1.28, CI 1.26–1.30) and PCI (0.7% vs. 0.3%; RR 2.57, CI 2.40–2.75). The angiography-to-PCI yield was correspondingly higher after PET than SPECT (15.0% vs. 5.9%), indicating that a greater proportion of angiograms performed after PET resulted in revascularization. On further analysis, major adverse cardiovascular events (MACE), defined as the composite of cardiovascular death, nonfatal myocardial infarction (ST-elevation or non-ST-elevation), and nonfatal stroke, occurred at similar rates after PET and SPECT MPI (4.7% vs. 5.1%; RR 0.92, 95% CI 0.78–1.09; *p* = 0.335).

## Discussion

In this propensity-matched analysis, PET MPI was associated with lower downstream ICA and higher procedural yield as measured by need for PCI compared with SPECT. Despite fewer referrals to ICA, PET was associated with a greater likelihood of subsequent PCI, suggesting improved selection of patients most likely to benefit from invasive evaluation rather than simple under-referral. Consistent with this interpretation, the composite rate of MACE events did not differ between modalities (4.7% vs. 5.1%; *p* = 0.335), indicating that the lower downstream angiographic utilization following PET was not achieved at the expense of an increase in myocardial infarction. Importantly, although sex-based differences in invasive utilization persisted, the relative advantages of PET over SPECT were observed consistently across both sexes.

These findings build upon and support prior work examining the impact of imaging modality on downstream care pathways and costs. Earlier analyses highlighted substantial inefficiencies in stress-testing-guided management, with a high proportion of patients undergoing ICA but relatively low rates of revascularization [3]. Subsequent data demonstrated that PET MPI was associated with lower downstream resource utilization compared with SPECT, suggesting that improved diagnostic performance may translate into more efficient care [4].

A randomized trial comparing PET and SPECT MPI demonstrated that although PET did not lead to an overall reduction in downstream testing or short-term clinical events, it was associated with improved concordance between ischemic risk assessment and subsequent invasive management, with higher rates of catheterization and revascularization among patients with high-risk PET findings and fewer invasive procedures among those with low-risk studies [5]. However, the trial was underpowered (161 patients per arm) to detect differences in clinical outcomes and conducted in a pre-selected population with established CAD. In addition, the investigators used attenuation-corrected SPECT, which may not reflect routine practice across all centers, may limit broader generalizability. Nonetheless, the findings support the concept that PET MPI enhances risk stratification and may facilitate more appropriate downstream utilization. Our findings are consistent with prior work by Pelletier-Galarneau et al., who demonstrated favorable downstream utilization patterns following PET MPI. In the present study, we extend these observations in a large multicenter real-world cohort and show that PET was associated not only with fewer downstream invasive coronary angiograms but also with a substantially higher angiography-to-PCI yield, supporting more efficient selection of patients for invasive evaluation [6].

Our study advances this literature in several important ways. First, by leveraging a large contemporary EHR-based network, we demonstrate that these associations persist in real-world practice at scale, beyond the confines of single-center or trial settings. Second, we explicitly examined procedural yield, providing evidence that reduced angiography following PET does not come at the expense of missed revascularization opportunities. Rather, PET appears to better align invasive evaluation with clinically actionable disease. Third, we show that the relative advantage of PET over SPECT is preserved across sexes. In keeping with this, the angiography-to-PCI yield was higher after PET than SPECT (15.0% vs. 5.9%). Because under-referral of patients with severe disease would be expected to lower rather than raise this yield, the lower angiography rate after PET is more consistent with selective, appropriate referral than with missed obstructive coronary disease. It is important to emphasize that our analysis characterizes downstream invasive utilization and procedural yield rather than the diagnostic accuracy of PET versus SPECT for obstructive CAD, since some patients with significant disease are appropriately managed medically or referred for surgical revascularization without PCI. For this reason, PCI was prespecified as the marker of procedural yield most directly downstream of the imaging-guided angiogram, whereas coronary artery bypass grafting which is comparatively infrequent in this population and is governed largely by heart-team and surgical considerations beyond the imaging pathway-was not evaluated and represents a direction for future dedicated analysis.

In contemporary value-based care frameworks, the economic impact of an imaging strategy should be assessed across the entire diagnostic pathway rather than determined solely by test acquisition cost. Therefore, our findings support our initial hypothesis that although PET MPI may involve higher upfront costs compared with SPECT, it may ultimately prove more cost-effective by mitigating cumulative downstream expenditures associated with invasive procedures.

This study has several limitations inherent to its observational design. Despite propensity-score matching, residual confounding may persist. TriNetX lacks data on ischemic burden, coronary anatomy, symptom severity, and physician decision-making that may influence ICA referral. Accordingly, the PET and SPECT cohorts could not be matched on pre-test probability of CAD directly, although the consistency of findings after matching available demographic and comorbidity variables argues against major imbalance.

In conclusion, among patients undergoing MPI, PET was associated with lower downstream ICA and higher procedural PCI yield compared with SPECT. These findings support a potential role for PET MPI in optimizing invasive coronary evaluation and improving efficiency of care in contemporary clinical practice of CAD.

## Data Availability

The data and analytic code used in this study can be made available on reasonable request to the corresponding author.
